# Correlation Analysis of the Transcriptome and Gut Microbiota in *Salmo trutta* Resistance to *Aeromonas salmonicida*

**DOI:** 10.3390/microorganisms12101983

**Published:** 2024-09-30

**Authors:** Shuaijie Sun, Jun Lv, Kuankuan Lei, Zhuangzhuang Wang, Wanliang Wang, Zhichao Li, Ming Li, Jianshe Zhou

**Affiliations:** 1 Institute of Fisheries Science, Xizang Academy of Agriculture and Animal Husbandry Sciences, Lhasa 850032, China; 15738816518@163.com (S.S.); lkk9992022@163.com (K.L.); zwangzhuang@163.com (Z.W.); qlxlsylzfyzx@163.com (W.W.); 2 College of Animal Science and Technology, Henan Agricultural University, Zhengzhou 450046, China; zhichaoli2014@outlook.com (Z.L.); liming@henau.edu.cn (M.L.); 3 Henan Academy of Fishery Sciences, Henan Academy of Agricultural Sciences, Zhengzhou 450044, China; 13838097113@163.com

**Keywords:** *Salmo trutta*, *Aeromonas salmonicida*, RNA-seq, 16S rRNA, resistance

## Abstract

*Aeromonas salmonicida* is a major pathogenic bacterium that poses a significant threat to salmonid fish. Yadong County, located in the Xizang Autonomous Region, is renowned for its characteristic industry of *Salmo trutta* aquaculture. In recent years, the outbreak of Bacterial Gill Disease (BGD) has led to substantial economic losses for *S. trutta* farmers. Our prior research identified *A. salmonicida* as one of the primary culprits behind BGD. To mitigate the impact of *A. salmonicida* on *S. trutta*, we conducted a comprehensive study aimed at identifying genes associated with resistance to *A. salmonicida*. This involved transcriptome sequencing and 16S rRNA sequencing of intestinal flora, providing valuable insights for the study of disease resistance in *S. trutta*. In this study, we identified 324 genera with 5171 ASVs in the susceptible group and 293 genera with 5669 ASVs in the resistant group. Notably, *Methylobacterium* and *Sphingomonas* were common bacteria present in the salmon’s gut, and their proportions remained relatively stable before and after infection. *Shewanella*, with its antagonistic relationship with Aeromonas, may play a crucial role in the salmon’s defense against *A. salmonicida*. Several related genes were identified, including *angptl4*, *cipcb*, *grasp*, *ccr9a*, *sulf1*, *mtmr11*, *B3GNT3*, *mt2*, *PLXDC1*, and *ank1b*.

## 1. Introduction

One of the foremost global challenges is to provide nutritious diets to populations with limited resources [1]. Aquaculture offers a promising solution to combat malnutrition and diet-related diseases [2]. Aquatic products are rich in essential nutrients of global significance, such as iron (Fe), zinc (Zn), calcium (Ca), vitamin A, and docosahexaenoic acid (DHA) [3]. However, the prevalence of diseases significantly impacts the survival of aquatic organisms and the quality of aquatic products [4]. *Aeromonas salmonicida*, a facultative gram-negative bacterium, primarily resides in water and represents one of the earliest recognized sources of infection in fish diseases [5]. It is chiefly responsible for conditions like fish furunculosis [6,7] and septicemia [8,9]. Nevertheless, there have been documented cases of infections spreading to other animals, including migratory birds [10], swine [11], and even humans [12,13]. Once *A. salmonicida* gains entry into fish hosts through the skin, intestines, or gills, it migrates to and colonizes vital organs like the head kidney, liver, spleen, and brain [9], thereby posing a substantial disease threat to fish.

*Salmo trutta*, commonly referred to as *Brown trout*, belongs to the Salmoniforme family within the Salmonidae fish group [14]. This species is renowned for its rich nutritional profile, delectable meat, absence of muscle spines, high protein content, and low cholesterol levels [15]. Cultivated *S. trutta* is considered a premium aquatic product and has earned recognition as a geographical indicator of agricultural products in China (http://www.anluyun.com/Home/Product/27301, accessed on 9 August 2024) and as an agricultural product with distinctive Chinese characteristics (http://www.moa.gov.cn/xw/zxfb/201912/t20191209_6332990.htm, accessed on 9 August 2024). *S. trutta* serves as one of the primary hosts for *A. salmonicida* [16] and is particularly susceptible to *A. salmonicida* infections, which can lead to disease outbreaks. These outbreaks have a profound impact on artificial breeding, resulting in significant fish mortality and substantial economic losses [17].

Gut microbiota plays a pivotal role in the overall health of fish as it can exert profound influences on various aspects, including the physiological functions of fish [18], growth performance [19], vitamin synthesis [20], nutrient absorption [21], metabolic processes [22], and immune responses [23]. Furthermore, it acts as a vital line of defense for fish in their battle against diseases [24,25]. Given the multifaceted roles of intestinal flora and its substantial fluctuations during aquaculture and across different stages of the aquaculture cycle [26], the composition and diversity of gut microbiota emerge as pivotal and informative biomarkers for assessing the health status and metabolic potential of fish [27,28,29]. Currently, there is a paucity of research on biomarkers for measuring the disease resistance of fish. Research on fish intestinal flora holds the promise of providing microbial markers for the identification and development of disease-resistant genetic lines and offers valuable insights and references for disease-resistant breeding strategies [30].

Furthermore, it is important to recognize that the resistance of fish to pathogen infections is an exceedingly intricate trait [31]. For instance, when considering *S. trutta*, it is evident that different individuals exhibit varying levels of resistance to *A. salmonicida*. Some *S. trutta* can not only survive bacterial outbreaks but also become lifelong asymptomatic carriers of the pathogen [16]. Importantly, this trait of disease resistance can be passed on through inheritance [32]. In the context of complex diseases, approaches like vaccination [33] and antibiotic treatment [34] have limitations, including limited coverage, high treatment costs, the potential for inducing antibiotic resistance [35], and leaving chemical residues in the environment. As a complementary strategy, disease-resistant breeding shows promise in enhancing the disease resistance of fish in aquaculture [36]. Understanding the resistance of fish to pathogen infections at the transcriptional level enables the rapid identification of antibacterial-related genes in fish, thereby contributing to the advancement of fish disease-resistant breeding efforts [37].

In our prior research, we successfully isolated and purified a strain of *A. salmonicida* from the diseased gills of *S. trutta* afflicted by bacterial gill disease. Subsequent artificial infection tests confirmed the pathogenic nature of this bacterium, while also revealing that some individuals within the *S. Trutta* population exhibited resistance to the pathogen. These findings present a valuable opportunity to investigate the distinctions between the intestinal flora and immune-related genes of resilient and susceptible *S. trutta* populations.

## 2. Materials and Methods

### 2.1. Sample Acquisition

In our previous research, we isolated and purified *A. salmonicida* and prepared a concentrated bacterial suspension at a concentration of 1 × 10^8^ CFU/mL. We then conducted an artificial infection experiment using 120 one-year-old *S. trutta*, with an average weight of (33.26 ± 8.11) g and a total length of (148.14 ± 12.32) mm. Each fish was intraperitoneally injected with 0.1 mL of the high-concentration bacterial suspension. The introduction of the bacteria led to severe pathological symptoms, ultimately resulting in the death of 98 *S. trutta* (81.67% of the total), categorizing them as the susceptible group (susceptible group). An additional 22 *S. trutta* (18.33%) survived the infection without displaying significant pathological symptoms, and these were designated as the resistant group (resistant group). We collected head kidney tissue and intestinal contents from both groups, rapidly froze them in liquid nitrogen, and stored them at −80℃ for preservation. Blood samples were also collected from both groups; serum was separated, according to the method of the Elisa kit (Meimian Co. Ltd., Yancheng, China); and the levels of three inflammation-related indicators, namely IgA (MM-1941O2), IL-1β(MM-0083O2), and TGF-β1 (MM-1829O2), were measured by a microplate reader (Thermo Scientific™ Multiskan™ FC, Thermo Fisher Scientific, Waltham, Massachusetts, the USA). Furthermore, gill and head kidney tissues were harvested from both groups, fixed with paraformaldehyde, and processed for histological analysis. Tissue sections were prepared and examined under a microscope (Nikon Eclipse ci, Nikon Precision Machinery Co., LTD, Shanghai, China) after hematoxylin and eosin (HE) staining [38].

### 2.2. Transcriptome Sequencing Analysis of Head Kidney Tissue

From the two groups of *S. trutta*, six head-kidney tissue samples were randomly selected. These samples were sent to “Shanghai Personal Biotechnology Co., Ltd. (Shanghai, China)” for transcriptome sequencing analysis. The total RNA was extracted from the kidney tissue using Trizol (TransGen Biotech, Beijing, China), and after quality inspection, the total RNA was reverse transcribed into cDNA and the original data were obtained using the Illumina sequencing platform. The initial step involved filtering the raw data. Cutadapt (v1.18) was used to remove the 3′ end linker, which had at least 10 bp overlap with the known linker (AGATCGGAAG), and a 20% base mismatch was allowed. At the same time, the reads with an average quality score lower than Q20 were removed. The clean data were obtained by the method described above. These clean data were then compared to the reference genome of *S. trutta* (Salmo_trutta.fSalTru1.1). Based on the comparison results, the expression levels of each gene were calculated. Subsequently, DESeq software (v1.24.1) was employed for further analysis of expression differences between the samples (|log2(FoldChange)| > 1, *p* < 0.05). Cluster analysis was performed using Pheatmap software (v1.0.12). To provide functional insights, GO annotations were carried out using Blast2GO software (v3.3.2), referencing the Gene Ontology database (http://geneontology.org/ accessed on 20 September 2024) established by the Gene Ontology Consortium. Additionally, KEGG annotations were generated using KAAS website (http://www.genome.jp/tools/kaas/ accessed on 20 September 2024), utilizing the Kyoto Encyclopedia of Genes and Genomes (http://www.kegg.jp/ accessed on 20 September 2024).

### 2.3. 16S rRNA Omics Analysis of Intestinal Flora

From the two groups of collected intestinal contents, we selected 12 samples for head kidney transcriptome sequencing and randomly added 5 samples to each group for 16S rRNA sequencing analysis. The total DNA was extracted from the intestinal content samples using a fecal genomic DNA extraction kit (Zepin Technology, Hangzhou, China). After passing quality control assessments, paired-end sequencing of community DNA fragments was performed on the Illumina platform. Subsequently, the library and samples were organized based on index and barcode information derived from the initial sequence data. The barcode sequences were removed, and the sequences were denoised using the QIIME2 dada2 analysis pipeline. We used “qiime cutadapt trim-paired” to remove primer fragments of sequences and discard sequences without matching primers. Then, DADA2 is called through “qiime dada2 denoise-paired” for quality control, denoising, splicing, and de-mosaicism. Taxonomic classification was performed using the Greengenes2 database [39]. To evaluate the alpha diversity within each sample, we assessed the distribution of Amplicon Sequence Variants (ASVs). At the ASV level, we calculated the distance matrix for each sample and measured the differences and statistical significance in beta diversity between different samples. Additionally, we explored species abundance composition diversity among different samples at the taxonomic level and attempted to identify marker species.

### 2.4. Prediction and Analysis of the Functional Potential of Intestinal Flora

Using the PICRUSt2 software (v2.5.3) [40], we embarked on a multi-step process to analyze the 16S rRNA gene sequences of known microbial genomes. Initially, these sequences were aligned, and an evolutionary tree was constructed, enabling the inference of gene functional profiles for common ancestors. A new evolutionary tree was then established by aligning the 16S rRNA feature sequences with reference sequences. To predict the closest species of the feature sequences, we employed the Castor hidden state prediction algorithm. This prediction was made based on the copy number of the gene family corresponding to the reference sequence within the evolutionary tree. Subsequently, we determined the copy number of gene families for each sample in accordance with the abundance of their characteristic sequences. Finally, we ‘mapped’ these gene families to various databases, including MetaCyc, KEGG, and COG databases. This allowed us to infer the presence of metabolic pathways using MinPath, ultimately providing us with data on the abundance of these metabolic pathways within each sample.

### 2.5. Correlation Analysis between Host Gene and Intestinal Flora

Utilizing the R software package “vegan (v2.6-8)”, we computed the Bray-Curtis distance matrix for both the transcriptome data and flora abundance data. Subsequently, we conducted a Mantel test for statistical analysis using the QIIME2 software [41], performing replacement tests on the samples (999 iterations). This allowed us to determine the statistical significance, denoted by the *p*-value, of the similarity between the transcriptome data and the composition of the microbiome. We employed the distance matrix obtained from the Mantel test to execute Procrustes analysis on the PCoA sequencing results of the transcriptome data and intestinal flora data [42], comprehensively assessing the correlation between the two datasets using M² and *p* values (derived from 999 permutation tests). By using the R software package “ade4 (v1.7-22)”, we conducted PCA at the genus level on the diversity composition profiles and transcriptome data. Subsequently, we performed CIA (coinertia analysis) [43]. The Spearman rank correlation coefficient between the transcriptome data and flora abundance was calculated using Mothur software (v1.48.1). Based on the correlation coefficient matrix and verification results, we created a heatmap using R software. Additionally, an association network was constructed using information where |rho| > 0.6 and *p*-value < 0.05 and visualized using Cytoscape software (v3.9.1) [44,45].

## 3. Results

### 3.1. Certain Phenotypic Distinctions Exist between the Group That Is Resistant and the One That Is Susceptible among S. trutta

**Figure 1:**
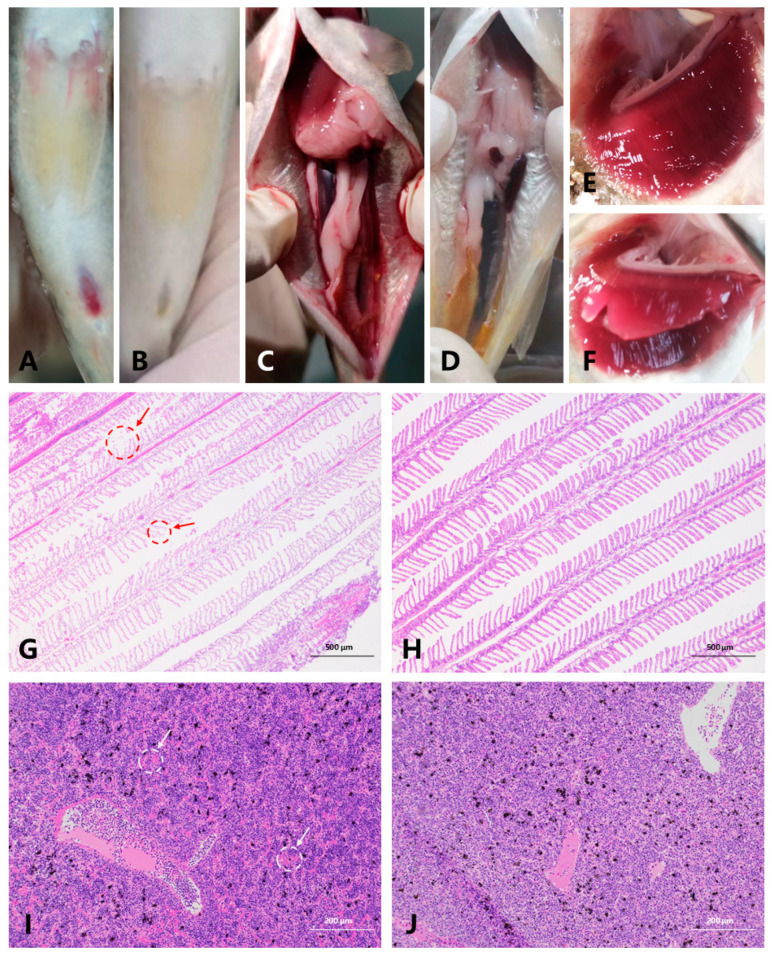
Phenotypic differences between susceptible and resistant groups. (**A**) Abdomen of the susceptible group; (**B**) Resistance group abdomen; (**C**) Susceptible group abdominal cavity; (**D**) Resistance group abdominal cavity; (**E**) Susceptible group gill; (**F**) Resistant branchial group; (**G**) Gill tissue sections of the susceptible group (40×), the arrow indicates the gill filaments were severely damaged; (**H**) Gill tissue sections of the resistant group (40×); (**I**) Sections of head kidney tissue in the susceptible group (100×), at the point indicated by the arrow, inflammatory cell infiltration was increased in the head kidney tissue; (**J**) Head kidney tissue sections of the resistant group (100×).

**Figure 2:**
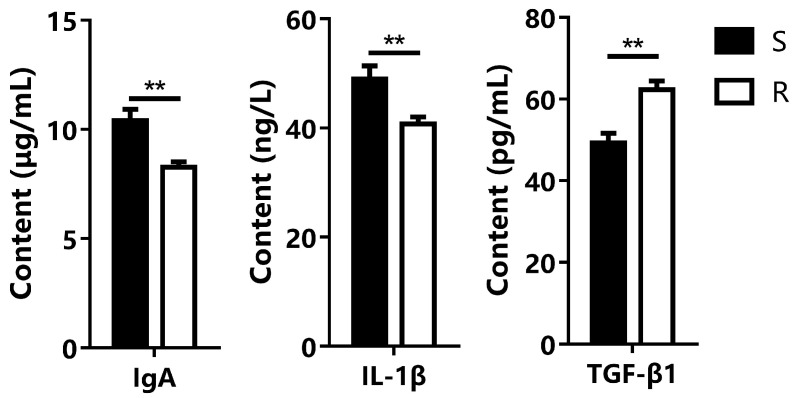
Differences in serum indexes related to inflammation between resistant and susceptible groups. Note: The “**” in the figure indicates that the difference is extremely significant.

After infection with *A. salmonicida*, notable differences were observed between the susceptible and resistant groups of *S. trutta*. In the resistant group, there was no bleeding or lesion in the excretion hole (Figure 1B), abdominal cavity (Figure 1D), or gill (Figure 1F) of *S. trutta*. In the susceptible group, evident bleeding was observed at the base of the stomach and excretion hole (Figure 1A). Upon abdominal cavity examination, multiple areas displayed bleeding (Figure 1C). Gill observations revealed extensive bleeding, bruising, ulceration, and damage to the gill filaments in the susceptible group (Figure 1E). When comparing gill tissue, it became apparent that the gill filaments in the susceptible group became thinner, disorganized, and damaged (Figure 1G). Moreover, the head kidney tissue exhibited congestion and an increase in inflammatory cell infiltration (Figure 1I). Furthermore, an analysis of three serum markers, IgA, IL-1β, and TGF-β1, showed significant differences. In comparison to the susceptible group, the resistant group exhibited a highly significant decrease in the levels of IgA and IL-1β in serum (*p* < 0.01), while the content of TGF-β1 was significantly increased (*p* < 0.01) (Figure 2).

### 3.2. Differences in Transcriptome Sequencing of the Head Kidney between the Resistant and Susceptible Groups

**Figure 3:**
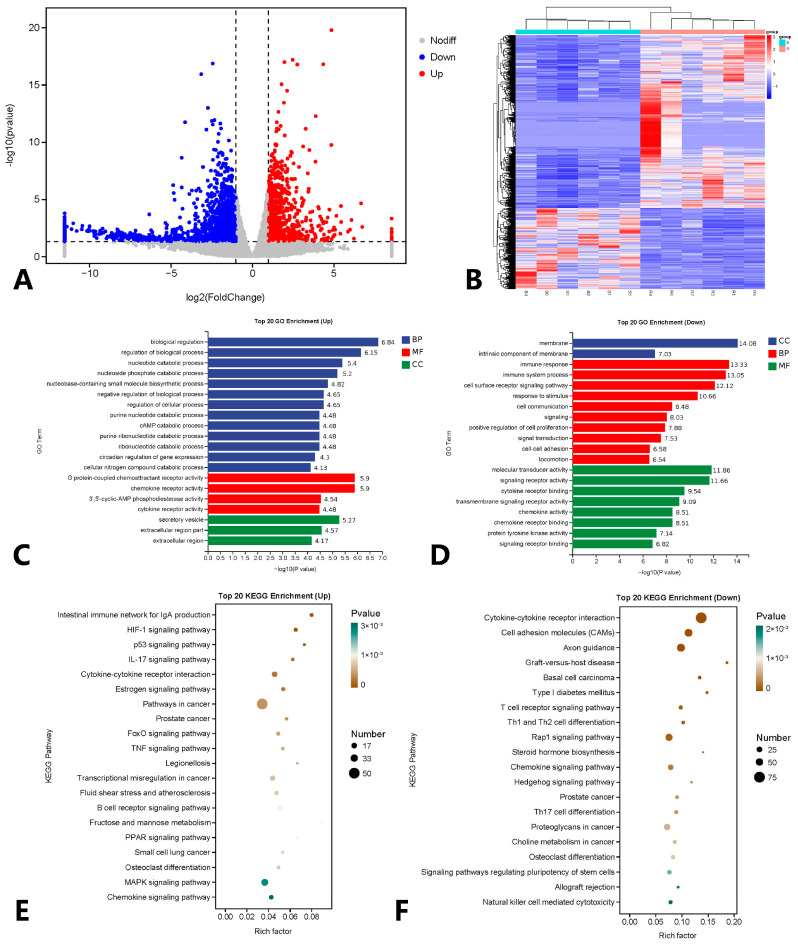
Distribution and related functions of differentially expressed genes in susceptible and resistant groups. (**A**) Volcano map of different genes between resistant group and susceptible group; (**B**) Heat maps of different genes between resistant and susceptible groups; (**C**) GO enrichment analysis of differentially up-regulated genes between resistant and susceptible groups; (**D**) GO enrichment analysis of differentially down-regulated genes between resistant and susceptible groups; (**E**) KEGG enrichment analysis of differentially upregulated genes between resistant and susceptible groups; (**F**) KEGG enrichment analysis of differentially down-regulated genes between resistant and susceptible groups.

Upon comparing gene expression profiles between the resistant and susceptible groups, we identified a total of 2528 differentially expressed genes (DEGs) (|log2(FoldChange)| > 1, *p* < 0.05). Among these, 807 genes were up-regulated and 1721 genes were down-regulated (Figure 3A,B). Notably, these up-regulated and down-regulated genes exhibited enrichments in distinct GO terms (Figure 3C,D) and KEGG functional classes (Figure 3E,F). Within the top 20 GO terms, we found that 3 terms in Cellular Component (CC), 13 in Biological Process (BP), and 4 in Molecular Function (MF) were upregulated in the resistant group. The top 10 up-regulated differential genes included *ddit4*, *rgs13*, *GLUL*, *ero1a*, *atf3*, *si:ch73-141c7.1*, *stk35*, *gng12a*, *ATP6V0E1*, and *mmp9*. These up-regulated genes in the resistant group were significantly associated with processes like the intestinal immune network for IgA production, HIF-1 signaling pathway, p53 signaling pathway, pathways in cancer, and fructose and mannose metabolism, along with various other metabolic pathways. In contrast, the down-regulated genes in the resistance group comprised 2 in CC, 10 in BP, and 8 in MF among the top 20 GO terms. The top 10 down-regulated differential genes included *ENSSTUG00000048521*, *gja5b*, *ENSSTUG00000034071*, *lck*, *themis*, *plxnb1a*, *ENSSTUG00000041667*, *ENSSTUG00000051057*, *CSF1R*, and *negaly6*. These down-regulated genes in the resistant group were notably associated with processes such as cytokine–cytokine receptor interaction, axon guidance, graft-versus-host disease, steroid hormone biosynthesis, and signaling pathways regulating pluripotency of stem cells, among other metabolic pathways.

### 3.3. Differences in the Composition of Intestinal Flora between the Resistant Group and the Susceptible Group, along with the Prediction of Their Functional Roles

**Figure 4:**
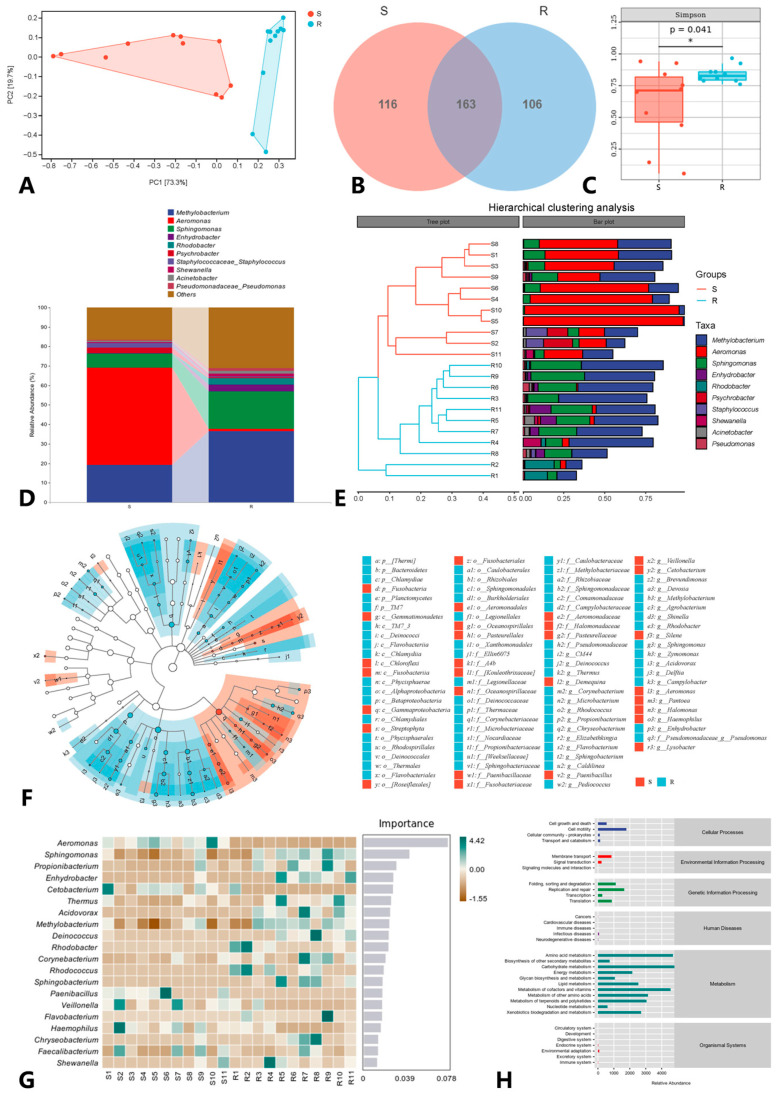
Intestinal microflora composition and differences between groups of *S. trutta*. (**A**) PCA map between resistant group and susceptible group (generic level); (**B**) Venn diagram between resistant group and susceptible group (generic level); (**C**) Simpson index results in α diversity; (**D**) Taxonomic composition analysis between resistant group and susceptible group (genus level); (**E**) Hierarchical cluster analysis between resistant group and susceptible group (generic level); (**F**) Taxonomic cladogram of species analyzed by LEfSe; (**G**) Random forest analysis; (**H**) Statistical map of metabolic pathway abundance. * significant differences between the resistant and susceptible groups (*p* > 0.001).

The composition of intestinal flora also exhibited marked distinctions between the resistant and susceptible groups. When the samples from both groups were separately grouped (Figure 4A), a comprehensive examination at the genus level revealed a classification of 385 genera in the intestinal flora, with 163 genera shared between the resistant and susceptible groups. The susceptible group had 116 unique genera, while the resistant group possessed 106 unique genera (Figure 4B). Alpha diversity analysis did not highlight significant differences between the resistant and susceptible groups (*p* > 0.001) (Figure 4C). Stratigraphical cluster analysis of β diversity at the genus level identified the top 5 bacteria in terms of abundance for the susceptible group as *Aeromonas*, *Methylobacterium*, *Sphingomonas*, *Psychrobacter*, and *Staphylococcus*. In the resistant group, the top five abundant bacteria were *Methylobacterium*, *Sphingomonas*, *Enhydrobacter*, *Rhodobacter*, and *Deefgea* (Figure 4D,E). LEfSe analysis pinpointed the bacteria with the most significant differences in the susceptible group, primarily concentrated in *Gammaproteobacteria*. In contrast, the resistant group exhibited pronounced differences in *Alphaproteobacteria*, Betaproteobacteria, and Bacteroidetes (Figure 4F). Conducting random forest analysis at the genus level, we determined that the top five bacteria in terms of comprehensive importance were *Aeromonas*, *Sphingomonas*, *Propionibacterium*, *Enhydrobacter*, and *Cetobacterium* (Figure 4G). Functional prediction analysis revealed that microbial functions were predominantly concentrated in the category of Metabolism. The primary metabolic pathways encompassed carbohydrate metabolism, amino acid metabolism, and metabolism of cofactors and vitamins. Furthermore, these microorganisms’ functions exhibited enrichments in broader categories, including cellular processes, genetic information processing, and human diseases (Figure 4H).

### 3.4. Correlation Analysis between Host Genes and the Composition of the Intestinal Flora

**Figure 5:**
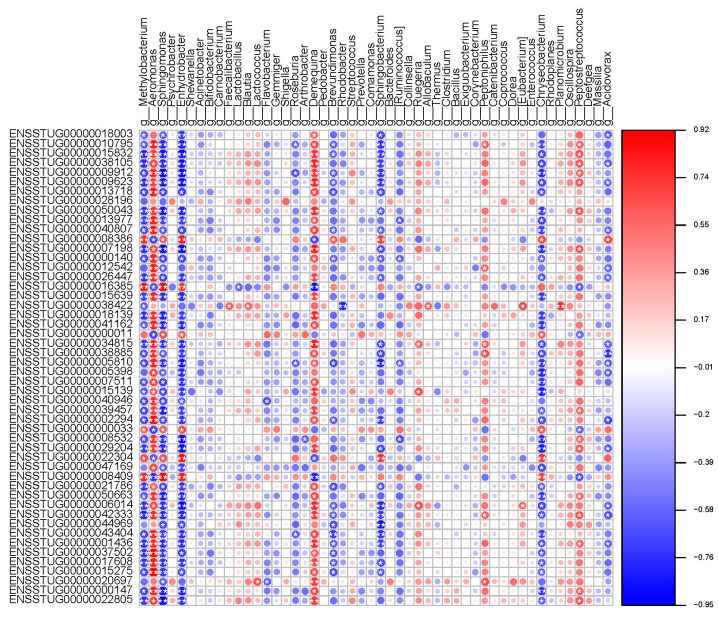
Association analysis between flora species and transcriptome differential genes. Correlation heat map. Note: If the correlation is positive, it is shown in red; if it is negative, it is shown in blue; The depth of color and the size of the circle indicate the strength of the correlation. * marked the “species-differential gene” with significant association (*p* < 0.05). ** in the figure indicates that the difference is extremely significant.

According to the Mantel test, the correlation coefficient between transcriptome data and flora abundance was r = 0.61, the *p*-value was 0.003, which is greater than 0.001 and less than 0.01, and the difference was significant. The M^2^ value of transcriptome data and microflora composition data obtained by Procrustes analysis was 0.51, the *p*-value was 0.005, which is greater than 0.001 and less than 0.01, and the difference was significant. In conclusion, the correlation test between transcriptome data and microflora composition data had high statistical significance and reliable results. The association analysis of host genes and gut microbiota uncovered 13784 pairs of “species-metabolites” with significant associations, including 807 genes and 306 genera. Most of the top 10 candidate genes that have a gene-level relationship with bacterial communities are involved in the immune response. The top 10 genera identified by pairing with gene frequencies are *Campylobacter*, *Aeromonas*, *Enhydrobacter*, *Methylobacterium*, *Propionibacterium*, *Pediococcus*, *Demequina*, *Sphingomonas*, *Chryseobacterium*, and *Paracoccus* (Figure 5). The top three abundant bacteria are *Methylobacterium*, *Aeromonas*, and *Sphingomonas*.

## 4. Discussion

### 4.1. Effects of A. salmonicida on S. trutta

Symptoms of *A. salmonicida* infection in fish are diverse. Following infection with *A. salmonicida*, carp display an array of symptoms, including abnormal swimming, lethargy, abdominal fluid accumulation, excretion hole bleeding, and fin bleeding [46]. Histological sections reveal a loss of tubular structure in the kidney, accompanied by white blood cell infiltration. In the gills, there is pronounced lamellar fusion, along with leukocyte infiltration, telangiectasia, and lamellar epithelial cell proliferation [46]. *A. salmonicida*, being the primary pathogen responsible for furunculosis in salmon trout, induces various clinical manifestations depending on factors such as fish health status, age, species, and environmental conditions, particularly water temperature. Acute furunculosis, generally seen in young fish up to 1 year of age, leads to systemic bacterial septicemia and high mortality within a short span of two to three days. In contrast, chronic furunculosis occurs in older fish, typically subadults and adults, and is characterized by boils in the muscles. Research indicates that *A. salmonicida*-infected rainbow trout tend to colonize in the dorsal fin, pectoral fin, and gills before infiltrating internal organs like the digestive system, spleen, or kidney and subsequently being excreted through the anus. The infection route appears to be linked to epithelial barrier damage, such as erosion or ulcers, to which the bacteria can attach and infiltrate host tissue.

In contrast, the susceptible group, after infection with *A. salmonicida*, exhibited multiple instances of bleeding in the abdominal cavity and intestine, along with ulceration at the ends of the gill filaments. Histologically, the gill filaments became thinner, and the tissue sustained damage. Additionally, congestion and inflammatory cell infiltration in the head and kidney were heightened, consistent with previous findings. Importantly, significant differences were observed in three serum indexes between the two groups (*p* < 0.01). Immunoglobulin A (IgA), as the principal immunoglobulin isotype produced by the intestinal immune system, plays a pivotal role in establishing a mutually beneficial host-bacterial relationship [47,48]. Interleukin-1β (IL-1β), a key mediator of the inflammatory response [49], increased in serum, indicating an intensified immune response. The rise in transforming growth factor β1 (TGF-β1), often termed a “Jack of all trades, master of everything,” in serum suggests an enhanced body resistance. TGF-β1 is essential for the development and maturation of immune cells in adult organisms, maintaining immune tolerance and homeostasis and regulating all aspects of the immune response [50]. The above results provide directions for subsequent research. We could explore which transcription factors affect the expression of serum immune markers [51] and possibly discover the mechanism of resistance of *S. trutta* to *A. salmonicida*. In summary, the impact of this bacterium on the fish’s physiology is multifaceted. In severe cases, it can not only lead to a significant compromise of the fish’s immune system, resulting in immune system failure, but can also affect the fish’s circulatory and respiratory systems, ultimately leading to the demise of the fish.

### 4.2. The Correlation between Differential Immune Genes and Metabolic Pathways in Relation to the Resistance of S. trutta to A. salmonicida

In this transcriptome sequencing analysis, it is noteworthy that the top 10 signaling pathways differentiating between the resistant and susceptible groups all bear significance for the health, disease, and immunity of the organism. The chemokine signaling pathway and T cell receptor signaling pathway, in particular, are intricately tied to the immune system of organisms. These two signaling pathways share the presence of six co-existing differential genes: *pik3r2*, *pik3r5*, *nfkbiab*, *ENSSTUG00000007836*, *ENSSTUG00000003555*, and *nfkbiaa*. Among these genes, there is a strong likelihood of them playing pivotal roles in the body’s immune response. Notably, *pik3r2* and *pik3r5* belong to the same gene family. Research by Liu et al. has indicated that *pik3r2* functions as a tumor-driving factor with elevated expression in most tumors, and its expression is linked to the degree of immune invasion across various tumors [52]. Furthermore, Liu et al. found that inhibiting the expression of *pik3r5* can regulate the AKT/mTOR signaling pathway, promoting epithelial–mesenchymal transformation and carcinogenic autophagy [53]. Both nfkbiab and nfkbiaa belong to the same gene family and are associated with the expression of NF-κB inhibitor α analogs. NF-κB, a transcription factor discovered three decades ago, plays a pivotal role in gene induction in various cellular responses, particularly within the immune system [54]. *ENSSTUG00000007836* and *ENSSTUG00000003555* are both linked to phosphoinositol-3 kinase (PI3K). Xu et al. demonstrated that the activation of the PI3K signal transduction pathway can enhance T cell immunity in the body [55]. Compagno et al. found that cytidine deaminase (AID) is a B-cell-specific enzyme, and the phosphatidylinositol 3-kinase δ (PI3Kδ) pathway regulates AID by inhibiting its expression in B cells [56]. These pathways have significant implications for the immune system and immune response in the body.

### 4.3. Relationship between Intestinal Flora and Resistance of S. trutta to A. salmonicida

The difference in intestinal flora between the susceptible group and the resistant group at the genus level is marked by a significantly higher proportion of Aeromonas in the susceptible group compared to the resistant group, whereas the proportion of *Shevanella* is significantly lower in the susceptible group as opposed to the resistant group. Upon comparing individuals within the two groups, it is evident that in the resistant group, those individuals with a higher content of Aeromonas also exhibit a higher content of *Shevanella*, and vice versa. However, such a phenomenon is notably absent in the susceptible group, where the majority of individuals have higher levels of Aeromonas. This observation suggests that the elevated proportion of Aeromonas in the susceptible group may be attributed to the group’s inability to resist the infection of *A. salmonicida*, leading to a higher Aeromonas concentration in their bodies. In contrast, most individuals in the resistant group have effectively resisted *A. salmonicida* infection, resulting in nearly negligible Aeromonas in their intestines. Still, some individuals in the resistant group do retain Aeromonas, and the content of *Shevanella* is also notably high. This might indicate a potential antagonistic relationship between *Shevanella* and *Aeromonas*. Previous research has identified 36 species of Aeromonas, at least 19 of which are considered to be potential pathogens for humans, leading to widespread infections [57,58]. *Shevanella*, on the other hand, is predominantly found in decaying fish, but in ocean environments, *Shevanella* species can produce eicosapentaenoic acid (EPA), particularly in permanently cold (e.g., polar or deep-sea) and/or high-pressure (deep-sea) habitats where EPA is generally beneficial under low/high-pressure conditions [59]. EPA is well known for its health benefits, and individuals of Asian East salmon with high *Shevanella* content do not exhibit abnormal pathological symptoms. Furthermore, both *Methylobacterium* and *Sphingomonas* show substantial abundance in the intestinal flora. *Methylobacterium* has significant biotechnological potential and has been studied for various applications, including bioreactors, polymer production, and plant growth promotion [60]. Species within the *Sphingomonas* genus have diverse functions, ranging from environmental pollution remediation to the production of highly beneficial plant hormones such as Gellan gum [61]. They likely play a pivotal role in maintaining the stability of the gut flora. But the intestine–immune system network is too complex. Future studies will require between-group analyses of the most relevant genetic circuits to determine associations.

### 4.4. The Role of Host Genes and Intestinal Flora in Helping S. trutta resist A. salmonicida

As the primary constituent of mucosal immunity, the gut microbiota serves as the foremost defensive barrier in aquatic animals, exerting a pivotal role in the regulation of the host’s immune system, overall health, and physiological processes [24,62,63]. Alterations in the gut microbiota are frequently associated with disease states [64,65,66]. The potential for enhancing fish health through the modulation of the gut microbiome using probiotics has garnered increasing attention [67,68,69,70,71]. To identify novel probiotics, it is imperative to obtain fundamental insights into their genetics and physiology, allowing for a more comprehensive understanding of their functionality and interactions with other gut microbes. The investigation of changes in the gut microbiota induced by diseases is of paramount importance for elucidating the onset and progression of illnesses and optimizing treatment strategies. Su et al. [72], in their study on the interaction between intestinal microbial communities and transcriptome profiles of the fourth generation of selected and wild Yellow River carp, employed Pearson correlation analysis to analyze a total of 2,892 pairs of genes (245) and bacterial genera (256). The majority of these identified associations were mapped to the realms of the immune system, bacterial communities, and cell differentiation processes. Fuess et al. conducted an analysis of the correlation between the composition of intestinal microbiota and the expression of immune genes in three-spine Acanthus [73]. They identified 15 microbial families that displayed a high degree of correlation with host gene expression, all of which were closely linked to the host’s expression of immune genes and associated processes. In a study by Zhou et al. investigating the resistance of gut microbes to vibriosis in Cyglossus semisulosus, it was found that the gut microbiota may modulate host immune homeostasis and inflammation through the microbial–gut–immune axis [29]. The combination of gut microbes and host genes as biomarkers demonstrated the ability to accurately distinguish between resistant and susceptible families. These collective studies substantiate the close connection between host immunity and the composition of the gut microbiome.

## 5. Conclusions

In this study, we delved into the intricate interplay between host genes and the microbiome of salmonid fish during their infection and resistance to *A. salmonicida*. Our investigation revealed noteworthy disparities in host gene expression and gut microbiota characteristics between the resistant and susceptible groups. Our findings underscore the pivotal role of the intestinal microbiota in regulating the expression of differentially expressed genes (DEGs), with notable up-regulation of DEGs such as *ddit4*, *rgs13*, *GLUL*, *ero1a*, and *atf3*, alongside down-regulation of *ENSSTUG00000048521*, *gja5b*, *ENSSTUG00000034071*, *lck*, and *themis*. These alterations collectively contribute to a reduction in inflammation levels within the resistant group, bolstering their defense against *A. salmonicida*. Crucially, our study unravels the significance of host gene-microbiota interactions within immune signal transduction pathways, which play a pivotal role in regulating host immune homeostasis and inflammation levels. These interactions, in turn, have a profound impact on enhancing resistance to *A. salmonicida*. Furthermore, the amalgamation of markers, encompassing both gut microbiome composition and host functional characteristics, robustly discriminates between resistant and susceptible families. These markers are closely entwined with phenotypic characteristics, suggesting their applicability in selective breeding efforts to bolster the germplasm of *S. trutta* in aquaculture.

Our study not only provides a deeper understanding of the role of intestinal flora and its functions in countering *A. salmonicida* but also offers valuable insights for enhancing the disease resistance of *S. trutta*. The host gene-microbiota interactions unveiled in our research may serve as a foundation for the development and genetic modification of probiotics, ultimately elevating disease resistance and productivity in *S. trutta* aquaculture.

## Data Availability

The animal experiment in this study was carried out at the Yarlung Zangbo Fishery Resource Breeding Base in the Xizang Autonomous Region, which was approved by the Ethical Committee for Institute of Fisheries Science, Xizang Academy of Agriculture and Animal Husbandry Sciences (Approval No. 2021005).
